# Non-invasive geophysical methods for monitoring the shallow aquifer based on time-lapse electrical resistivity tomography, magnetic resonance sounding, and spontaneous potential methods

**DOI:** 10.1038/s41598-024-58062-2

**Published:** 2024-03-27

**Authors:** Kaitian Li, Jianbo Yan, Fan Li, Kai Lu, Yongpeng Yu, Yulin Li, Lin Zhang, Peng Wang, Zhenyu Li, Yancheng Yang, Jiawen Wang

**Affiliations:** 1 School of Geophysics and Geomatics, China University of Geosciences, Wuhan, 430074 China; 2 Coal Geology Bureau of Ningxia Hui Autonomous Region, Yinchuan, 750002 Ningxia China; 3 Faculty of Engineering, China University of Geosciences, Wuhan, 430074 China; 4 College of Geology and Environment, Xi’an University of Science and Technology, 58 Yanta Rd., Xi’an, 710054 Shaanxi China; 5 School of Future Technology, China University of Geosciences, Wuhan, 430074 China; 6 Chengdu Surveying Geotechnical Research Institute Co., Ltd. of MCC, Chengdu, 610023 China

**Keywords:** Shallow aquifer, Water-conducting fracture zone, Electrical resistivity tomography, Magnetic resonance sounding, Spontaneous potential, Environmental sciences, Engineering

## Abstract

The Ningdong coalfield has played a pivotal role in advancing local economic development and meeting national energy. Nevertheless, mining operations have engendered ecological challenges encompassing subterranean water depletion, land desertification, and ground subsidence, primarily stemming from the disruption of coal seam roof strata. Consequently, the local ecosystem has incurred substantial harm. Water-preserved coal mining presently constitutes the pivotal technology in mitigating this problem. The primary challenge of this technique lies in identifying critical aquifer layers and understanding the heights of water-conducting fracture zones. To obtain a precise comprehension of the seepage patterns within the upper coal seam aquifer during mining, delineate the extent of water-conducting fracture zones, non-invasive geophysical techniques such as time-lapse electrical resistivity tomography (TL-ERT), magnetic resonance sounding (MRS), and spontaneous potential (SP) have been employed to monitor alterations within the shallow coalfield’s aquifer throughout the mining process in the Ningdong coalfield. By conducting meticulous examinations of fluctuations in resistivity, moisture content, and self-potential within the superjacent strata during coal seam extraction, the predominant underground water infiltration strata were ascertained, concurrently enabling the estimation of the development elevation of water-conducting fracture zones. This outcome furnishes a geophysical underpinning for endeavors concerning local water-preserved coal mining and ecological rehabilitation.

## Introduction

Subterranean coal mining operations have led to a substantial depletion of groundwater resources. Statistical data indicate that coal mining in China annually disturbs nearly eight billion metric tons of groundwater^1^. Within the arid climatic conditions of China’s western mining regions, where the rate of evaporation surpasses rainfall by a factor of six, this depletion of invaluable water resources exerts a formidable strain on local water provisions serving daily human needs and ecological preservation. Attaining a symbiotic relationship between coal mining and water resource preservation is of utmost importance. The principle of “water-preserved coal mining” underscores the safeguarding of water resources throughout the coal extraction process to avert substantial subterranean water seepage and its subsequent repercussions on the ecological milieu^2^. Executing water-preserved coal mining necessitates the initial identification of pivotal aquifer strata, along with the determination of development areas and elevations of water-conducting fracture zones^3,4^. Following this, pertinent engineering measures are implemented to preclude groundwater depletion and alleviate inundation accidents in coal seams.

Geophysical methods, serving as non-destructive investigative tools, are primarily employed to examine and analyze subsurface earth structures, subterranean resources, and diverse geological phenomena. These methods provide efficient and cost-effective means of obtaining a wide range of physical parameters related to subsurface materials. Three non-destructive geophysical techniques, TL-ERT, MRS, and SP methods, have been employed to monitor fluctuations in underground water within the Ningdong coalfield. ERT is a geophysical technique grounded in variations in the electrical properties of subsurface materials. This method consists in transmitting a direct current into the ground via surface electrodes to examine fluctuations in subsurface electrical conductivity. ERT offers satisfactory spatial resolution and sensitivity, enabling detailed mapping of both 2D and 3D subsurface electrical structures^5^. In the past decade, ERT has been extensively utilized in diverse fields, including engineering–geological surveys^6,7^, hydrogeological studies^8,9^, geological hazard evaluations^10,11^, and cultural heritage conservation^12,13^. In the field of mining, ERT is frequently employed for the identification of acid mine drainage (AMD) preferential pathways in tailing ponds^14–16^. Additionally, it is utilized to visualize the internal structure of tailing ponds^17–19^ in order to evaluate their potential environmental risks. It has evolved into a standard instrument within these disciplines. Time-lapse ERT (TL-ERT), an extension of the conventional ERT approach, can depict alterations in resistivity across varying time intervals. Its primary application lies in the evaluation of near-surface hydrogeology and its frequent use in landslide monitoring and environmental ecology surveys^20,21^. Over the past decade, TL-ERT has been applied to mining waste monitoring^22–25^. Magnetic resonance sounding (MRS), a relatively new geophysical method, has experienced continuous expansion in its application domain over the past decade, primarily attributable to rapid technological progress and the enhancement of associated theoretical frameworks. Initially utilized for assessing water resources and imaging subsurface karst formations^26,27^, researchers have also documented its utilization in the preservation of cultural heritage^13^. MRS predominantly relies on the relaxation signals of hydrogen protons within subterranean water, yielding direct insights into the existence of subsurface water^28^. Using MRS to complement ERT is really promising to quantify properly moisture content from ERT. As solely from the ERT data, it would be difficult to distinguish whether alterations in resistivity are attributable to groundwater variations. For instance, low resistivity areas mapped by ERT could also be associated with higher pore fluid electrical conductivity. Compared with the aforementioned methods, the SP method represents an ancient form of passive-source geophysical exploration^29^. This method operates independently of external sources and can analyze the movement of subsurface water using natural electric fields. At present, the SP method finds extensive application in diverse environmental and engineering sectors associated with groundwater flow, including mineral exploration^30^, as well as environmental monitoring for issues such as dam leakage^31^ and the transport of subsurface groundwater pollutants^32^.

In this study, we utilized TL-ERT, MRS, and SP methods to monitor underground water variations throughout the mining process at the Ningdong coalfield. TL-ERT and SP surveys cover the entire mining face, thereby providing geophysical characteristics at a relatively broader scale than MRS. Additionally, MRS provides a more precise and local quantitative understanding. The result can be used to constraint and enhance the comprehension of TL-ERT and SP results. The primary objectives were to examine the seepage patterns within the shallow aquifer layer following the disruption of coal seam roof strata during mining, outline the zones where water-conducting fracture belts developed, and ascertain the elevations of these fracture belts. TL-ERT records variations in resistivity along the mining face direction, whereas MRS offers direct groundwater detection, thereby effectively indicating changes in moisture content. Furthermore, fluctuations in self-potential provide valuable insights into trends related to groundwater seepage. These three geophysical methods facilitate a comprehensive analysis of groundwater seepage patterns, yielding robust geophysical evidence to support the implementation of water-preserved coal mining practices in the vicinity.

## Study area and geological setting

**Figure 1:**
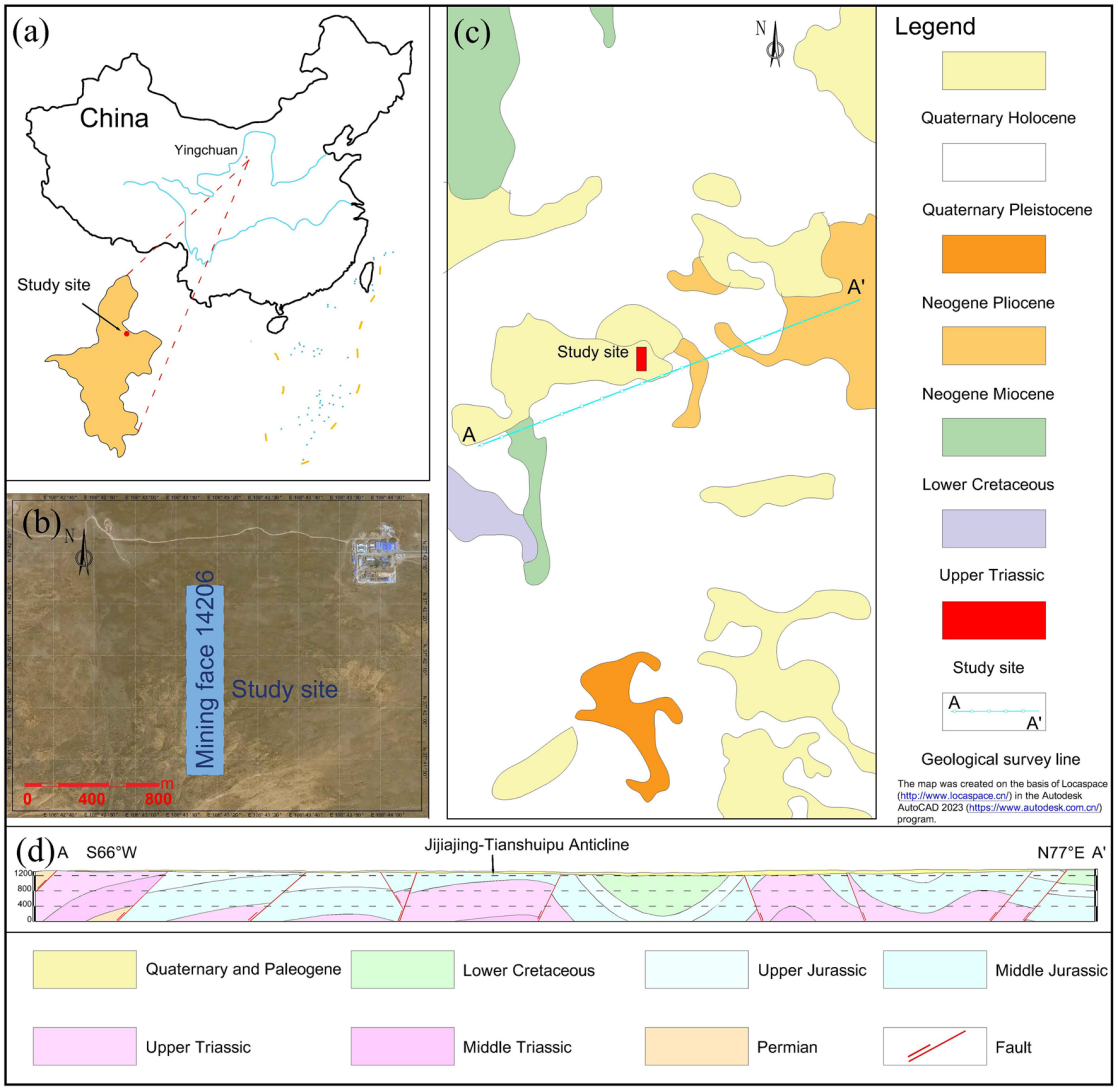
Geographical location and geology of the study site. (**a**) The study site is located in Ningxia Province, China; (**b**) satellite aerial view of the study site; (**c**) geological map of the study site; and (**d**) geological cross-section line AA’. The map was created on the basis of Locaspace (http://www.locaspace.cn/) in the Autodesk AutoCAD 2023 (https://www.autodesk.com.cn/) program.

The study site is situated in the northern region of the Jijiajing coalfield in Ningdong, located approximately 66 km southeast of Yinchuan City in Ningxia Province (Figure 1a). Its geographical coordinates span from an east longitude of 106°37′31″–106°44′15″ and a north latitude of 37°39′46″–37°47′02″. The area is situated in western inland China and has a semi-arid continental monsoon climate. The annual precipitation is usually below 300 mm, with the majority of rainfall concentrated in July, August, and September^33^. The topography of the study site (mining face 14206) consists of semi-desert low hills and relatively open terrain (Fig. 1b). The entire study area is blanketed by Quaternary sediments (Fig. 1c). This area is situated in the central portion of the north–south-oriented thrust fault zone on the eastern flank of the fold-and-thrust belt, at the southern boundary of the North China Platform and the western margin of the Ordos Basin. Specifically, this region represents a localized segment of the Jijiajing-Tianshuipu anticline (Fig. 1d). The structural orientations within the oilfield predominantly follow an NNW direction, with pronounced folding and faulting structures.

Based on borehole observations, the stratigraphy in this region encompasses various geological formations in chronological order: the Upper Triassic Shangtian group (T_3_s), Middle Jurassic Yan’an group (J_2_y), Middle Jurassic Zhiluo group (J_2z_), Upper Jurassic Anding group (J_3_a), Paleogene Eocene Qingshuiying group (E_3_q), and Quaternary (Q). Specifically, the Middle Jurassic Yan’an group in this area represents a coal-bearing stratum comprised of gray and gray–white fine-grained sandstones containing feldspar and quartz, gray and gray–black siltstones, mudstones, coal deposits, and a minor presence of aluminous claystone.

Considering the geological characteristics of sedimentary rocks and their hydraulic properties, the region can be primarily classified into two categories of groundwater: Cenozoic porous aquifers and porosity and fractures water in Mesozoic clastic rock. Cenozoic porous aquifers mainly comprise Quaternary aeolian sands, alluvial deposits, lacustrine sediments, and Paleogene subterranean gravel aquifers. Highly weathered rocks are commonly found near the upper boundary of the Mesozoic clastic rock basement, with weathering intensity increasing toward the surface. The pores, particularly fractures, within these weathered rocks exhibit significant development, occasionally establishing a network of aquifers in conjunction with the overlying Cenozoic aquifers, consequently bolstering their aquifer characteristics. In this region, the Middle Jurassic Yan’an group comprises coal-bearing rock sequences, with the upper strata of the coal seams housing sandstone aquifers characterized by a coarse lithology.

## Method

### Well camera

The inspection using the SKYJ-17 well camera was conducted on December 23, 2023, at the borehole located at the center of the mining face (Fig. 1a). This camera system employs both bottom and side cameras to capture images of the borehole wall, facilitating a comprehensive exploration of formation characteristics. The recorded depth of observation was 107 meters.

### Electrical resistivity tomography

**Figure 2:**
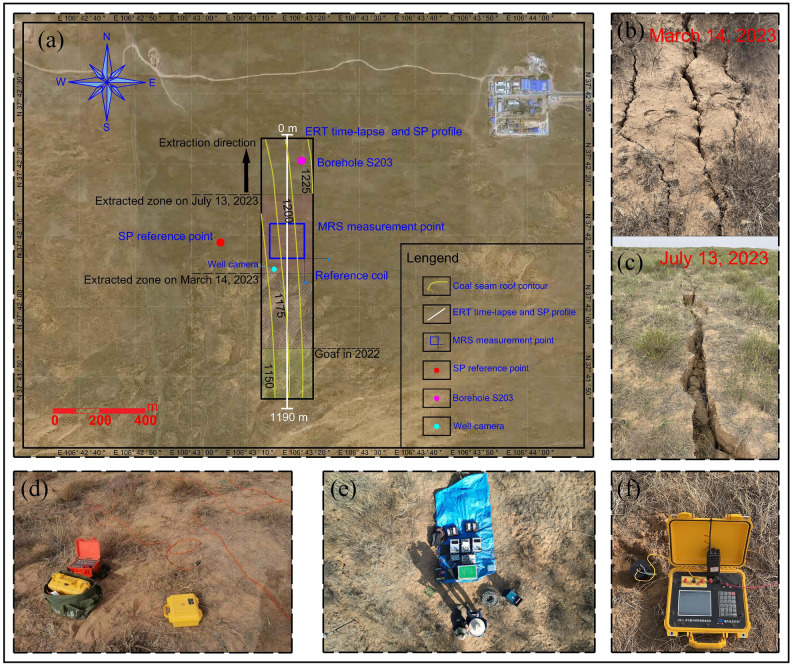
Arrangement of profile and point and on-site work photographs. (**a**) Arrangement of integrated geophysical methods and mining status; (**b**) surface cracks on 14 March 2023; (**c**) surface cracks on 13 July 2023; (**d**) image of ERT data collection; (**e**) image of MRS data collection; and (**f**) image of SP data collection.

As a non-destructive geophysical survey method, electrical resistivity tomography (ERT) enables the rapid acquisition of 2D and 3D electrical resistivity distributions within geological layers. In this ERT survey, the primary investigation focused on the coal mining face labeled as 14,206 within the study area (Fig. 2a). The thickness of the coal seam ranges from 1.17 to 4.20 m in this mining face, the average thickness is 3.35 m, and the mining height is 3.5 m. A DZD-8 direct current resistivity meter was employed for this purpose (Fig. 2d). Electrodes were spaced at 10 m intervals, resulting in a total of 120 electrodes arranged in a Wenner array. The precise positions of each electrode were recorded using real-time kinematic (RTK) technology. Two measurement sessions were conducted on 14 March 2023 and 15 July 2023. Excavation progressed from south to north, covering a distance of 1 km. In the initial measurement, the tunnel had advanced 600 m along the survey line, resulting in the emergence of vertical ground fissures perpendicular to the mining direction (Fig. 2b). In the subsequent measurement, the tunnel advanced an additional 240 m, resulting in the further expansion of ground fissures (Fig. 2c).

Time-lapse inversion was performed using the RES2DINV software (version 4.8.10), employing the least-squares inversion method using the following equation^34^.

1 $$\left[{J}_{i}^{T}{R}_{d}{J}_{i}+\left({\lambda }_{i}{F}_{R}+\alpha {M}^{T}{R}_{t}M\right)\right]\Delta {r}_{i}={J}_{i}^{T}{R}_{d}{g}_{i}-\left({\lambda }_{i}{F}_{R}+{\alpha }_{i}{M}^{T}{R}_{t}M\right){r}_{i-1}$$

In 2D inversion, ${F}_{R}={\alpha }_{x}{C}_{x}^{T}{R}_{m}{C}_{x}+{\alpha }_{z}{C}_{z}^{T}{R}_{m}{C}_{z}$. $\alpha$ is the temporal damping factor. ${C}_{x}$ and ${C}_{z}$ are the roughness matrices. $J$ is the Jacobian matrix. $r$ is the resistivity model for all the time series. $R$ is the weighting matrix. M is difference matrix applied across the time models. $g$ is the data misfit. Since the prevailing geological formations in this region primarily comprise sedimentary layers, a filtering ratio of 0.5 was employed to enhance the layering visualization. Considering the geological strata exposed through borehole drilling, it is noteworthy that the sandstone in this area generally demonstrates strong cementation. The initial observation acted as the reference model, and the time-lapse profiles illustrated the changes in resistivity relative to the first inversion results obtained during the second observation. To evaluate the practical depth of investigation of field data sets, and to verify the reliability of the inversion results, we conducted a DOI (depth of investigation) analysis according to the following form^35^.

2 $${R}_{AB}=\frac{{q}_{A}\left(x,z\right)-{q}_{B}\left(x,z\right)}{{q}_{A}-{q}_{B}}$$

Using two distinct reference resistivity values to invert the same data is the basic concept of the DOI analysis. The inversion results converge to the same resistivity value for regions with good data constraints, while for regions with poor data constraints, the inversion results are only similar to the reference resistivity. ${q}_{A}$ and ${q}_{B}$ represent two distinct reference resistivity values. ${q}_{A}\left(x,z\right)$ and ${q}_{B}\left(x,z\right)$ represent the inverted resistivity values under the reference resistivity of ${q}_{A}$ and ${q}_{B}$, respectively. ${R}_{AB}$ is the DOI index.

### Magnetic resonance sounding

The MRS method was conducted on 15 March 2023 and 16 July 2023 at the same location (Fig. 2e). The NUMIS^POLY^ instrument was employed, which is a state-of-the-art, high-sensitivity surface nuclear magnetic resonance water detection system manufactured by the French company IRIS. A 150 m, single-turn loop was used for both excitation and reception, while the closest high-voltage power line was situated approximately 2 km away. To enhance the signal-to-noise ratio, two reference coils, each with a side length of 10 m, were employed. Before conducting MRS measurements, it was essential to assess the local geomagnetic field. We conducted measurements of the magnetic field strength at the four corners and the coil’s center point, ensuring that variations did not exceed 10 nT. Subsequently, we computed the corresponding weighted average values, yielding a final magnetic field strength of 55,022.1 nT. The methodology necessitated the introduction of an alternating electromagnetic field with a frequency matching the precession frequency of hydrogen protons in groundwater, commonly known as the Larmor frequency ${f}_{L}$, and this is calculated using Eq. (3):

3 $${f}_{L}=\gamma \left|{B}_{0}\right|/2\pi$$

where $\gamma$ is the gyromagnetic ratio, and ${B}_{0}$ is the strength of the geomagnetic field.

For a single measurement, a total of 16 pulse moments were employed, and each pulse moment was stacked 64 times. The data inversion utilized the Occam inversion method^36,37^, yielding a histogram distribution of the water content down to a depth of 150 m below the surface.

### Spontaneous Potential

SP measurements were conducted on two distinct dates: 13 March 2023 and 14 July 2023. The SP method utilized a 10 m electrode spacing and employed a pair of non-polarizing Pb–PbCl_2_ electrodes to measure self-potential variations along the mining face. Each non-polarizing electrode was placed at the same positions as the electrodes used in the ERT method. Observations were conducted using the potential measurement method. For both observations, the reference electrode N was consistently positioned outside the mining workface where the natural potential remained stable (Fig. 2a). The measurement electrode M functioned as the active electrode and was employed to measure the potential difference relative to the reference point at each measurement point along the profile. To reduce the impact of the natural electrical current and electromagnetic field at the surface, both the reference and measurement electrodes were situated in 20 cm deep excavated pits. Upon completing potential observations along the entire profile, the M electrode was placed in the same location as the reference electrode to record the final potential difference and time. These data were used to correct for electrode potential drift during the measurements. The DZD-8 instrument (Figure 2f) was employed for self-potential measurements, boasting an accuracy of less than 1 µV, thereby ensuring measurement precision. The data inversion utilized the Genetic Algorithm (GA) inversion method^38^ and the forward model^39^ is represented by Eq. (4):

4 $$V\left(x\right)=kln\left\{\frac{{\left[\left(x-{x}_{0}\right)-acos\alpha \right]}^{2}+{\left(h-asin\alpha \right)}^{2}}{{\left[\left(x-{x}_{0}\right)+acos\alpha \right]}^{2}+{\left(h+asin\alpha \right)}^{2}}\right\}$$

where $k=\frac{I\rho }{2\pi }$ is the polarization parameter, $I$ is the current density, $\rho$ is the resistivity, ${x}_{0}$ is the zero position of the anomaly, a is the half width and $\alpha$ is the inclination of the medium.

## Results and discussion

### Well camera results

**Figure 3:**
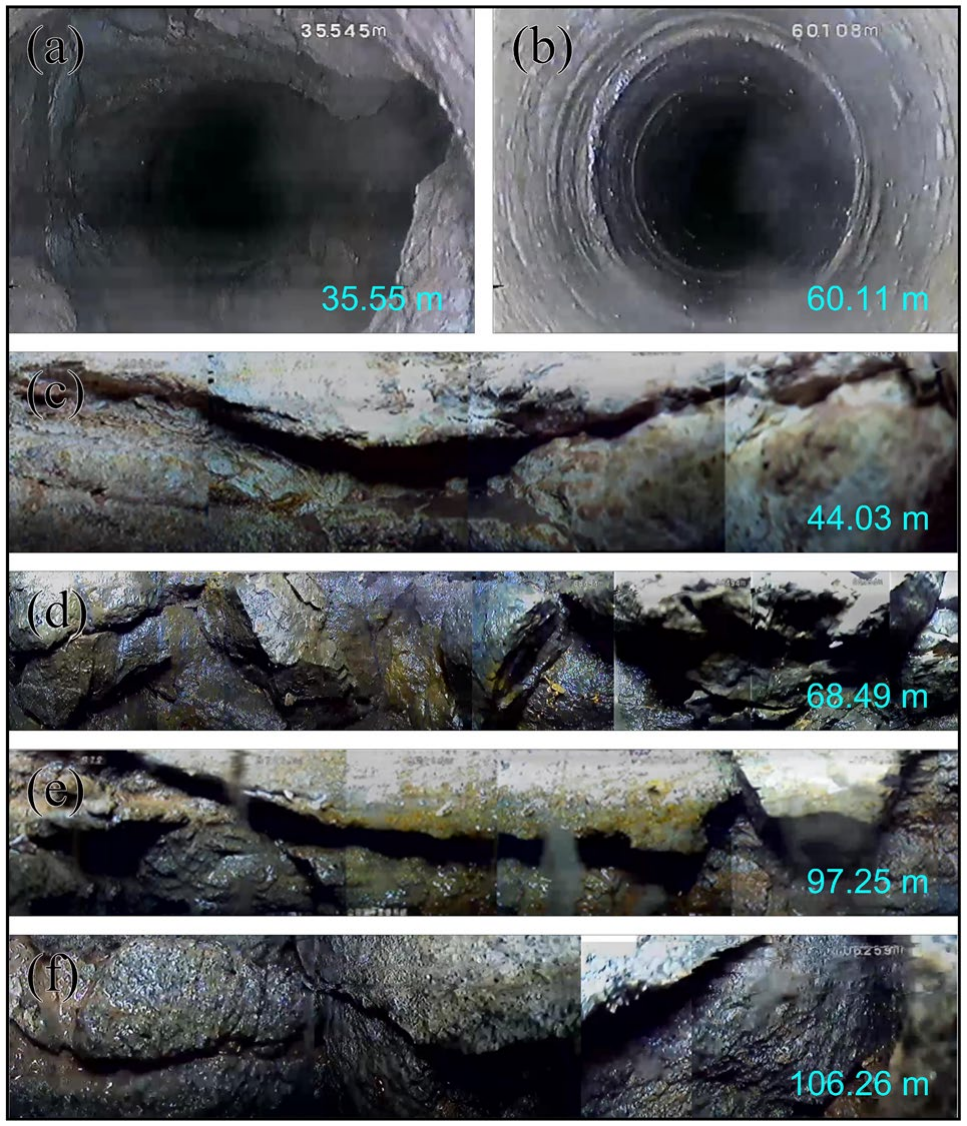
The stratigraphic images at different depth positions. (**a**) Weathered bedrock; (**b**) intact bedrock; (**c**) original fractures; (**d–f**) new fractures induced by mining operations.

Figure 3 illustrates images depicting various depths within the stratigraphic layers. Figure 3a presents the weathered bedrock layer, where no apparent water seepage features are observed above 35.55 meters. In Fig. 3b, the characteristics of the borehole wall are depicted under conditions of intact bedrock, exhibiting a smooth appearance. Additionally, Figure 3c displays the original fractures present within the weathered bedrock layer. Subsequent to coal mining activities, the overlying strata experience bending settlement and deformation, resulting in modifications to their internal structure. Local primary fractures incur deformational damage and generate numerous new fractures, notably including oblique fractures which exhibit good water conductivity, thereby facilitating pathways for underground water infiltration. Moreover, with increasing depth, the integrity of the rock layers deteriorates significantly (Fig. 3d–f).

### ERT results

**Figure 4:**
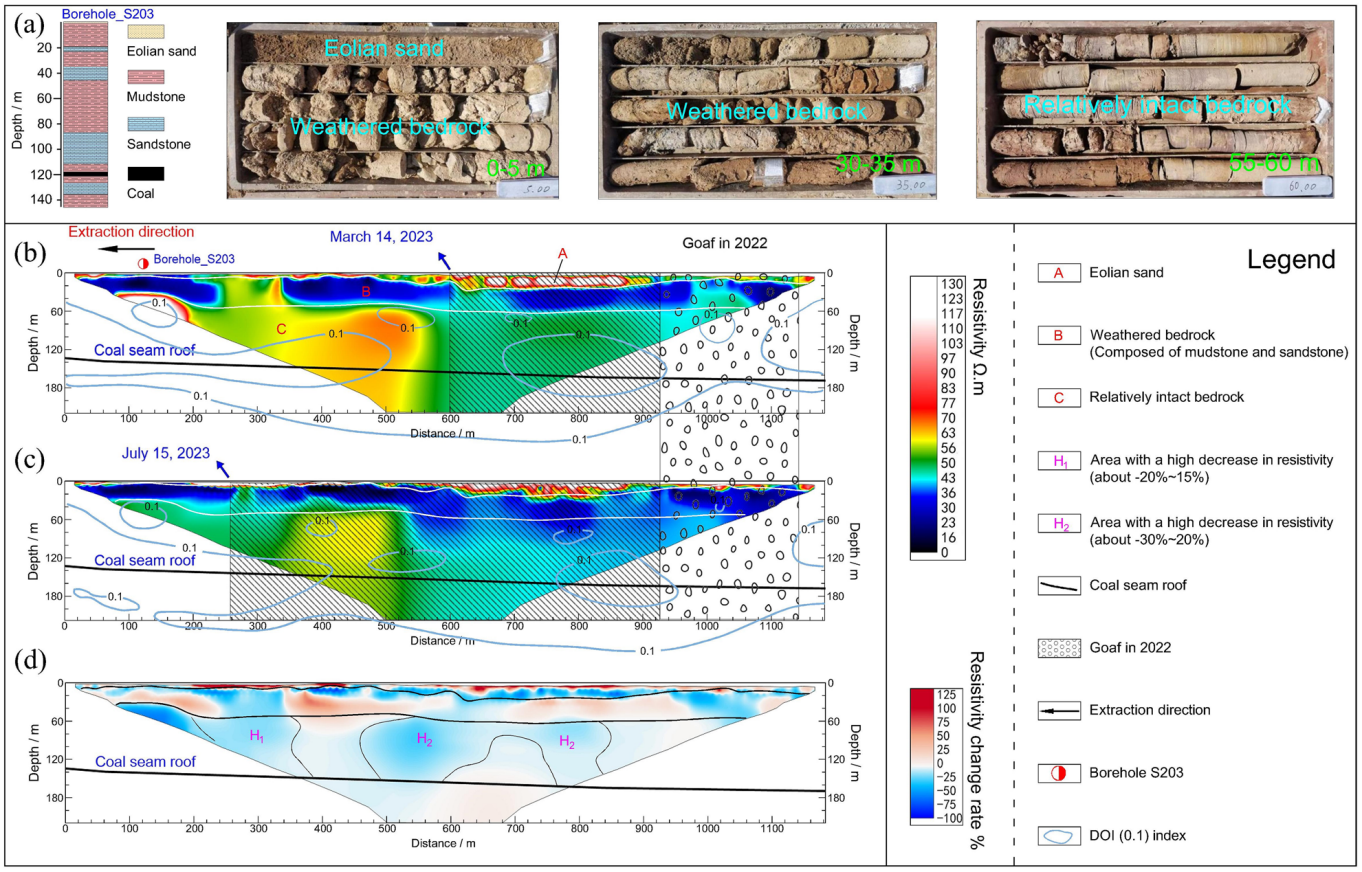
ERT results. (**a**) Photographs of core samples from borehole S203; (**b**) ERT profile was observed on 14 March 2023; (**c**) ERT profile was observed on 15 July 2023; and (**d**) time-lapse profile.

After eliminating data outliers and performing time-lapse inversion, two resistivity profiles from two separate observations were successfully acquired, along with profiles that illustrated the resistivity variations between these observations. Figure 4a shows the photographs of core samples from borehole S203. Figure 4b, c displays the resistivity profiles on 14 March 2023 and 15 July 2023. We projected the contour of the DOI value equaling 0.1 (a reasonably DOI value might be 0.1 or 0.2) onto each resistivity profile. The DOI values of most areas are lower than 0.1, reflecting the relatively high reliability of the resistivity profiles.

The geological structure within the study area is primarily categorized into three layers, and the findings are validated utilizing data from borehole S203 nearby the ERT profile (Fig. 4a). The first layer extends approximately 5–16 m below the surface, exhibiting resistivity values ranging from 80 to 120 Ω·m. This layer is interpreted as Quaternary aeolian sand, known for its comparatively high permeability. It contains stagnant water formed by rainfall infiltration (The moisture content is low, as MRS results have confirmed this), which results in an uneven lateral distribution of resistivity. The second layer is situated at depths ranging from 10 to 40 m and displays lower resistivity values, approximately between 30 and 40 Ω·m. This layer corresponds predominantly to the weathered zone of the bedrock, comprising interbedded sandstone and mudstone. Hydrological drilling findings suggest that the stable groundwater level in the region is approximately 35 m, indicating that the low-resistivity layer reflects the presence of water-bearing weathered bedrock. It is reasonable to infer that the lower resistivity boundary marks the interface between weathered bedrock and unaltered bedrock. The third layer is located at depths exceeding 40 m and exhibits relatively consistent resistivity values, roughly between 50 and 60 Ω·m. This layer is primarily composed of various grades of sandstone and mudstone. The depth of the coal seam roof on the north side is approximately 132 m, while on the south side it is about 160 m, with an average thickness of 3.35 m.

The coal seam is oriented from south to north in terms of the mining direction. In the initial measurement, the surveyed area extended from 600 to 950 m, encompassing the previously excavated region, whereas the segment spanning 950–1120 m denoted the mining void created in 2022. Within the third electrical layer, resistivity beyond the 600 m mark was significantly lower than in the shorter distance range. The high-resistivity anomaly observed at the profile’s left boundary can be attributed to data artifacts. By the time of the second measurement, mining had advanced to a distance of 260 m. In the third electrical layer, resistivity had generally decreased, resulting in an expanded low-resistivity zone and an overall resistivity reduction between 5 and 10 Ω·m. Figure 4d illustrates the time-lapse profile, depicting variations relative to the first measurement. The upper section of the first electrical layer indicates an increase in resistivity, while the lower part exhibits a decrease. This phenomenon may be attributed to seasonal rainfall causing waterlogging in the upper layer of the aeolian sand stratum. In the second electrical layer, the resistivity exhibited an overall increase of 10–20%. However, within the ranges of 260–320 m and 600–700 m, there was an approximate 10 to 20% decrease in resistivity. The third electrical layer displayed an overall reduction of 5–10%, with specific areas showing a 30% resistivity decrease. Notably, regions with more pronounced resistivity reductions, denoted as H1 and H2, suggest the development of water-conducting fracture zones ascending during the mining process. Groundwater within the weathered bedrock layer likely infiltrated downward along these water-conducting fracture zones and converged in the regions labeled H1 and H2.

### MRS results

**Figure 5:**
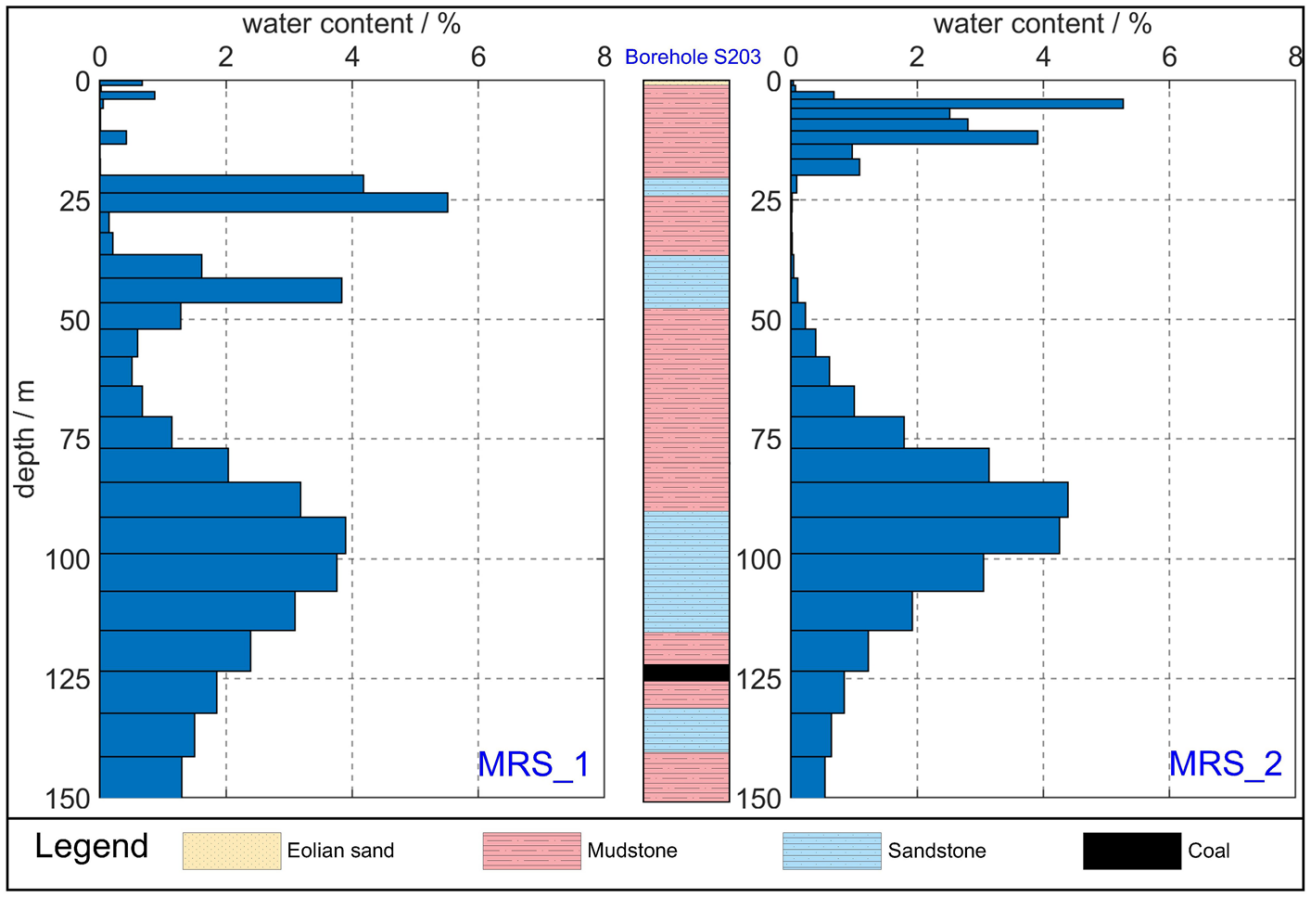
MRS results. MRS_1 was observed on 14 March 2023. MRS_2 was observed on 15 July 2023.

The MRS measurement points were centrally positioned along the ERT profile at a distance of 460 m. Data were acquired during two distinct observation sessions on 14 March 2023 and 15 July 2023. The inversion results produced a water content histogram spanning depths from 0 to 150 m below the surface. These measurement points were denoted as MRS_1 and MRS_2 (Fig. 5). The findings of the initial observation suggest that the subsurface is predominantly composed of three aquifer layers. The water content histogram indicates that the first aquifer layer lies at a depth range of 20–27 m, exhibiting a water content ranging from approximately 4.2–5.5%. The second aquifer layer is situated roughly between depths of 36 and 46 m, with a water content varying from 1.6 to 3.8%. According to information revealed by borehole 203, both of these water layers are situated within the weathered bedrock and correspond to the pore and fracture water within the sandstone layer. The third aquifer layer is positioned at an estimated depth of 85 to 115 m, displaying a water content ranging from 2.0 to 3.9%. This layer primarily comprises pore-pressure water within the sandstone formations.

**Figure 6:**
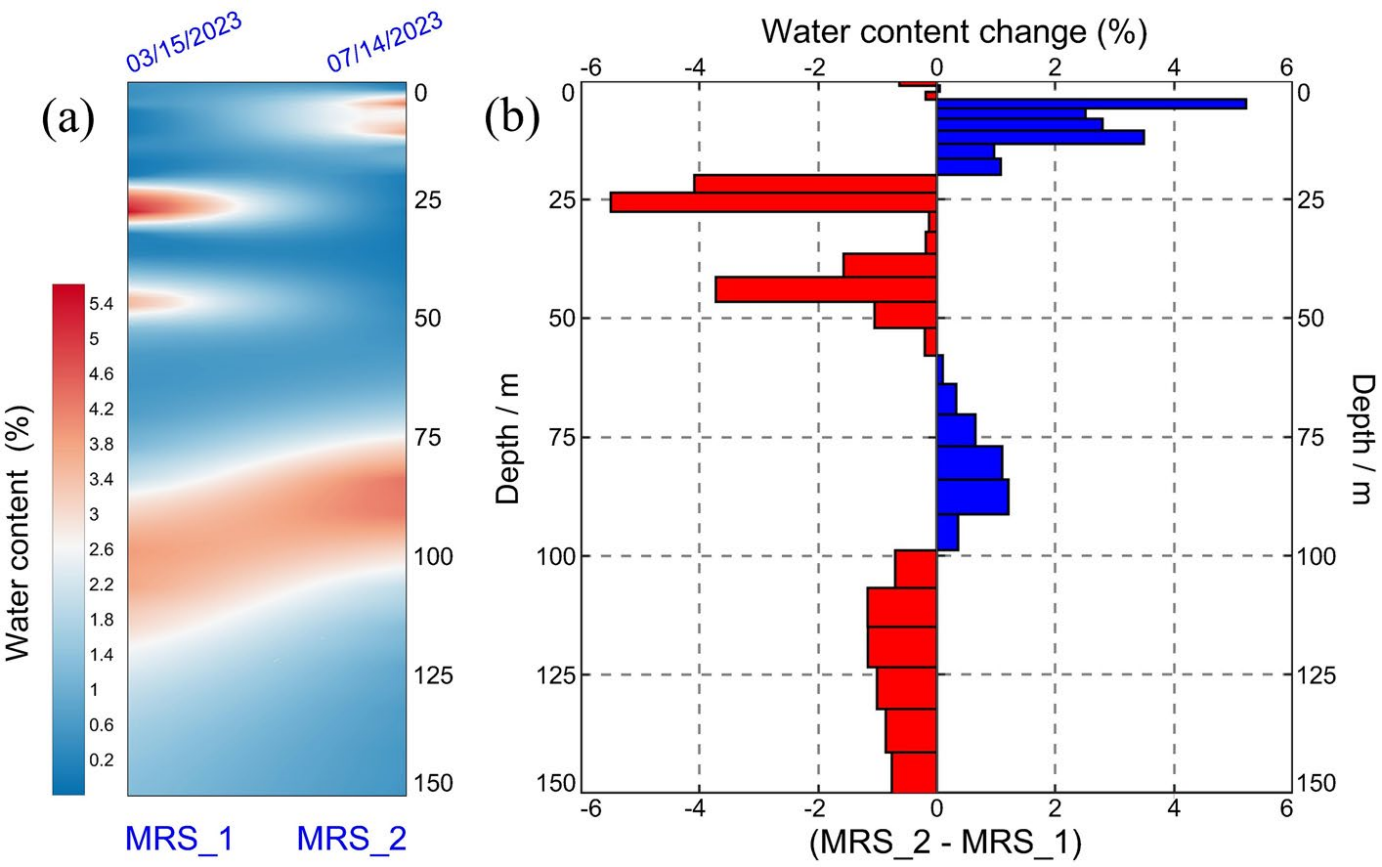
(**a**) Contour map of the water content for two observations; (**b**) differences in water content values.

Figure 6a shows the contour map of the water content for two observations. Figure 6b shows the differences in water content values. The results from the second observation reveal the subsurface’s division into two distinct aquifer layers, marked by shifts in the primary aquifer layer’s depth. However, the sandstone layers within the weathered bedrock exhibit a notable absence of water. The initial shallow aquifer layer is concentrated at depths ranging from 4 to 13 m, with a water content ranging from approximately 2.5–5.2%. Given that the second observation occurred during the rainy season in the study area, the heightened water content in the shallow aquifer layer can be largely attributed to precipitation. The secondary aquifer layer is located at depths spanning from 77 to 106 m, with a water content estimated to be approximately 3.1–4.4%. Overall, the water content in the range of 70 to 100 m has increased by approximately 1.6%, while the water content below 100m has decreased (Fig. 6b). It can be reasonably inferred that throughout the mining process, water-conducting fracture zones within the roof strata of the coal seam become interconnected with localized weathered bedrock layers. Consequently, this interconnection leads to the downward seepage of groundwater from the upper weathered bedrock through the water-conducting fracture zones, causing an increase in the water content of the coal seam roof.

### SP results

**Figure 7:**
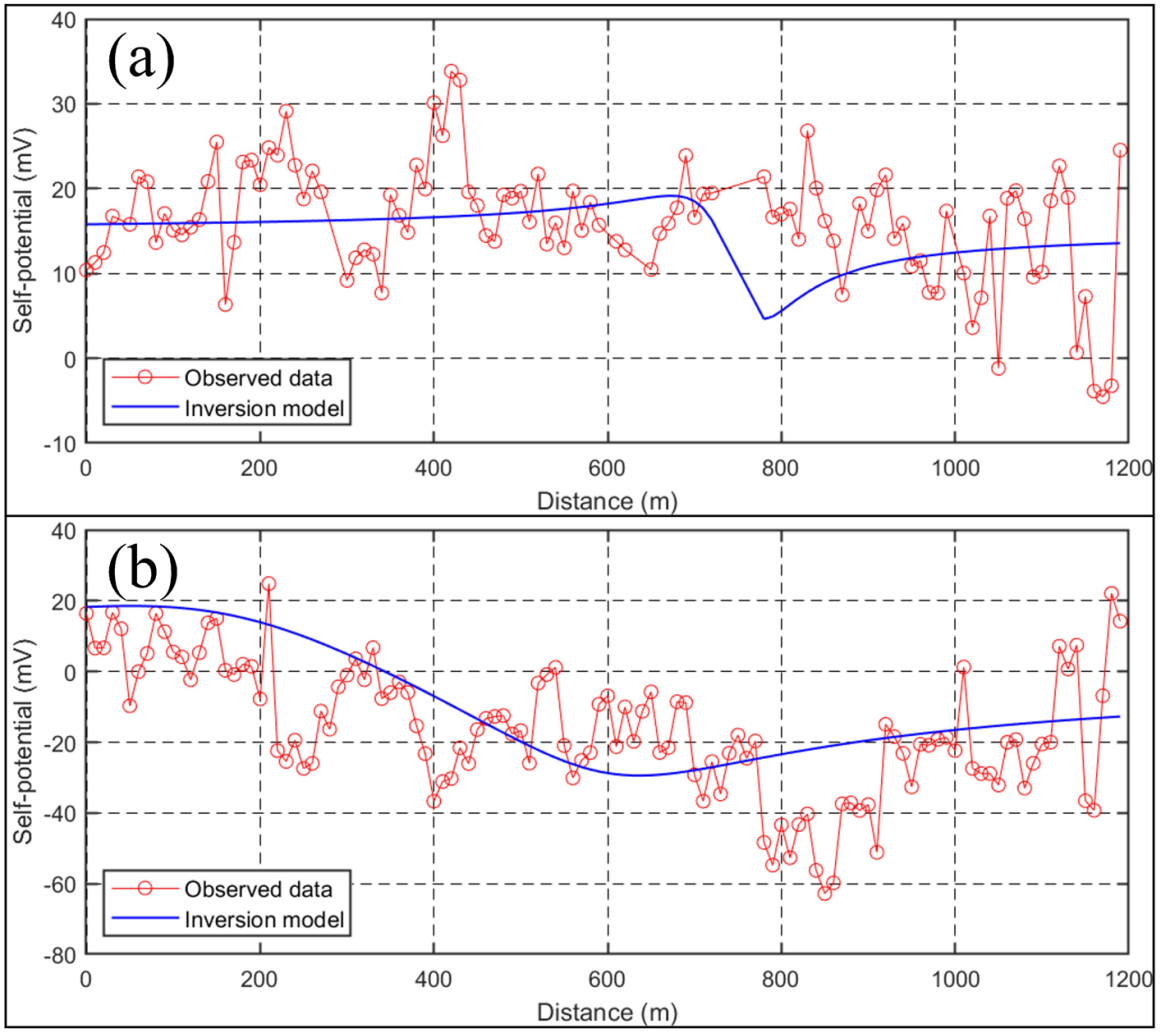
(**a**) The result of the first SP measurement; (**b**) the result of the second SP measurement. The red line with dots represents the self-potential values, while the blue line represents the inversion model.

There are power facilities and other coal mining equipment in the vicinity of the study area. Although certain data points exhibit relatively stable readings throughout the measurements, the possibility of interference cannot be disregarded. Consequently, outliers with large negative values, were eliminated. Figure 7a presents the initial findings. The results of the initial observation show self-potential values ranging between −4.53 and 33.84 mV, with an average of 18.32 mV and a variance of 47.74 mV. The inversion model demonstrated a satisfactory alignment with the observed data (Fig. 7a), after 100 iterations, yielding an iteration error of 0.98%. The findings indicate that the SP potential values are mainly positive. The estimated model parameters (Table 1) indicate that the center of the anomaly is located at a surface distance of 661.7 m, with a depth of 40.58 m, and a half-width of 4.90 m. This suggests that the region possibly served as a primary zone for groundwater infiltration during the initial observation.

**Table 1:** Estimated model parameters using Genetic Algorithm (GA).

	Estimated model parameters					RMS error
	$k{\text{ (m)}}$	$x_{0} {\text{ (m)}}$	$z{\text{ (m)}}$	$\alpha { (}^\circ {)}$	$a{\text{ (m)}}$	
SP_1	40.59	661.7	40.58	20.78	4.90	0.98
SP_2	26.71	380.1	42.85	40.44	286.98	0.57

Figure 7b presents the second findings. The results of the second observation reveal self-potential values ranging between −62.6 and 24.8 mV, with an average of −16.04 mV and a variance of 18.36 mV. The inversion model demonstrated a satisfactory alignment with the observed data, after 100 iterations, yielding an iteration error of 0.57%. In contrast with the first observation, these values decrease overall. The estimated model parameters (Table 1) indicate that the center of the anomaly is located at a surface distance of 380.1 m, with a depth of 42.85 m, and a half-width of 286.98 m. This suggests that the mining activities in the coal seam have significantly disrupted the roof bedrock, resulting in the development of fractures that establish connections with the shallow aquifer layer. Consequently, groundwater infiltration has increased.

### Discussion

Ground-truth data for the study site, obtained through direct observation using well camera, provides insight into the development of stratigraphic fractures. These fractures induced by mining activities may serve as vertical conduits for groundwater flow. During the excavation process, data on resistivity, water content, and self-potential values in different time periods were acquired in the study area through two sets of observations using three distinct geophysical methods. Based on the observations from well camera and geophysical results, the following inferences can be made. During the initial observation, the resistivity profile displayed clearer geological layering, with more pronounced resistivity distinctions in regions not impacted by mining activities (mileage < 600 m). In these undisturbed regions, the overall resistivity of the intact bedrock layers exceeded that of the mining-affected areas. With the SP method, the estimated model parameters indicate that the anomaly center is located at a depth of 40.58 m and a surface distance of 661.7 m, located within the weathered bedrock layer.

**Figure 8:**
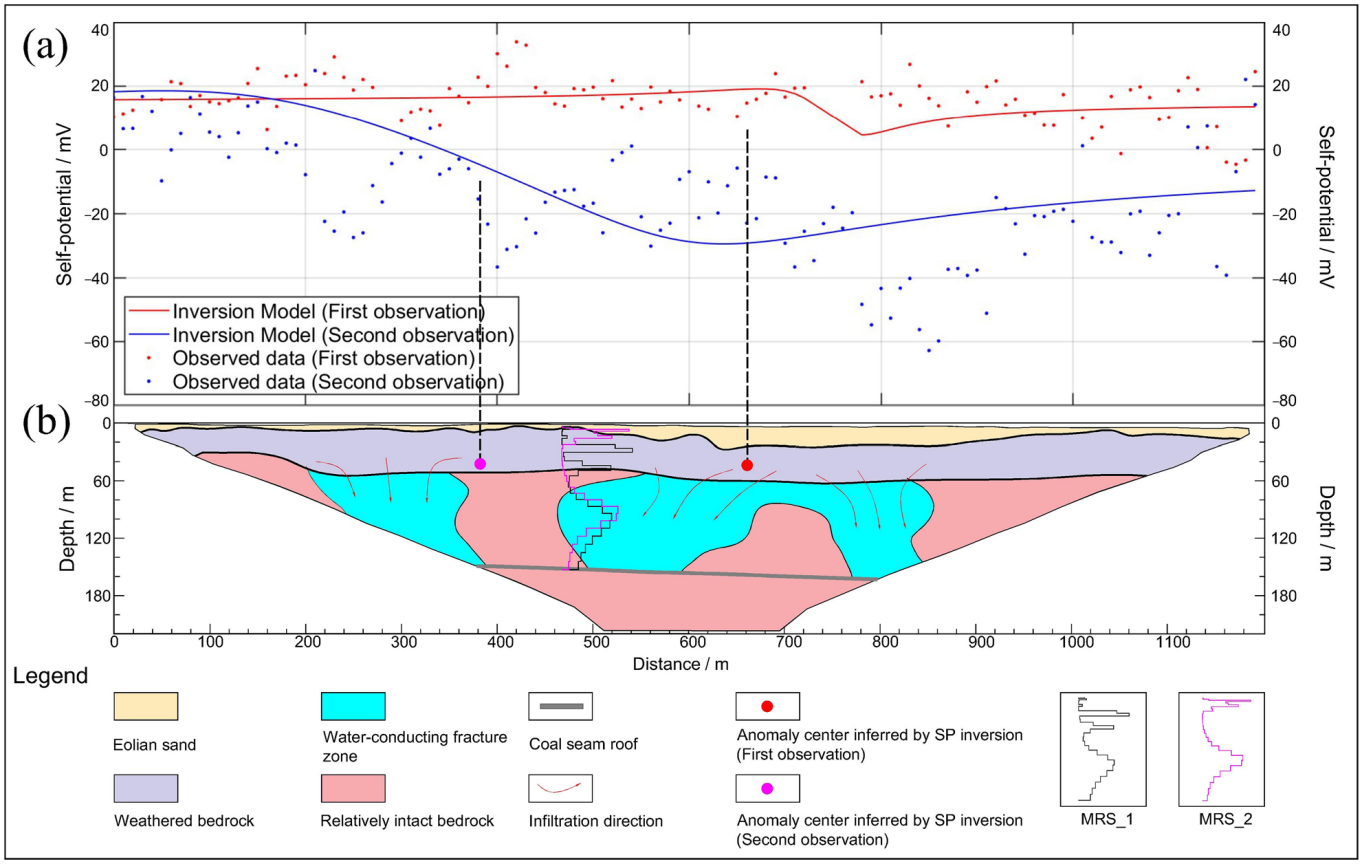
Integrated interpretation map of the study site. (**a**) Results of two SP measurements and the inversion model; (**b**)inferred stratigraphic structure and distribution of water-conducting fracture zone.

In the second observation, the study area experienced significant mining-related disruptions, leading to an increase in the resistivity of the weathered bedrock layer and a general decline in the resistivity of the underlying intact bedrock. Furthermore, the MRS findings indicated heightened moisture levels in the deeper subsurface strata (with the weathered bedrock layer exhibiting an almost negligible water content). The time-lapse resistivity profiles unveiled localized connections between the weathered bedrock layer and the lower intact bedrock at specific distances, potentially serving as permeable conduits for upward seepage, consequently forming preferential flow channels. In contrast with the initial SP observation, the second measurement predominantly revealed negative potential values. The estimated model parameters indicate that the anomaly center is located at a depth of 42.85 m and a half width of 286.98 m. The results of both observations exhibit an overall descending trend (Fig. 8a), signifying a comprehensive downward migration of groundwater above the mining face. This occurrence aligns with the augmented moisture content in the lower aquifer layer and the reduced resistivity in the bedrock layer.

Based on the results of the time-lapse ERT, MRS, and SP measurements, a quantitative understanding of the changes in the upper aquifer layers in the mining face area was achieved. The stratigraphic structure within the mining face region was delineated, the distribution of water-conducting fracture zones was inferred, and the groundwater seepage patterns in the upper coal seam during the coal mining process were reconstructed (Fig. 8b). As mining progressed and goaves were created, the overlying strata of the coal seam continually fractured and subsided. According to the disclosures from borehole S203, a 24 m thick layer of medium-grain sandstone exists approximately 6 m above the coal seam roof, which is the most rigid layer in the study area. After the development of fractures in this sandstone layer, as the mining face expanded gradually, these fractures extended into the weathered bedrock layer, establishing connectivity between the shallow aquifer layer and the underlying bedrock. This led to the gradual downward percolation of groundwater in the weathered bedrock layer, resulting in an increase in the moisture content in deeper layers and a decrease in resistivity. Simultaneously, due to a reduction in the moisture content in the shallow weathered bedrock layer, its resistivity increased. The time-lapse ERT profiles effectively characterize this process. The areas with reduced resistivity can be considered as zones of fracture development within the intact bedrock layer, exhibiting a “saddle-shaped” morphology.

In this study, the inversion outcomes obtained using the MRS method serve as a valuable complement to the interpretation of resistivity data. While the direct current resistivity method is less susceptible to electromagnetic interference and yields robust results, its applicability to assessing variations in subsurface water content is inherently ambiguous. Furthermore, the surfaces of clay particles exhibit notable electrical conductivity attributed to aluminum and magnesium ions. Within this study’s scope, variations in water content in the geological layers are considered the most influential factor. Despite being influenced by electromagnetic interference, the signal-to-noise ratio of the MRS inversion results is not exceptionally high (1.68 and 2.02, respectively), potentially introducing some bias to the subsurface water content estimation. However, MRS provides a direct estimation of groundwater changes, which is pivotal for comprehending groundwater dynamics during the mining process. Although the SP method cannot directly estimate specific physical parameters, alterations in self-potential serve as significant indicators of groundwater flow processes. One-dimensional SP inversion makes the assumption of a solitary subsurface anomaly. Within the model parameters of 1D-SP inversion, the parameter ‘z’ may signify the central point of primary groundwater infiltration, while ‘a’ denotes its reach. These estimates offer a simplification for intricate hydrological processes and are subject to certain constraints. Additionally, due to significant electrical interference in the vicinity of the study area, the SP results were subject to a certain degree of disruption (wherein the measured data did not exhibit satisfactory continuity), potentially resulting in some degree of bias or even misguidance. Incorporating the SP method introduced an additional dimension to validate the findings obtained using the other two methodologies.

## Conclusions

In this study, three non-invasive geophysical techniques, the TL-ERT, MRS, and SP methods, were employed to monitor the groundwater dynamics throughout the mining operations at the mining face 14206 of the Ningdong coalfield. After excavation, well camera observation was conducted, which serves as a significant supplement to the geophysical inferences. Time-lapse ERT inversion predominantly elucidates variations in subsurface resistivity across distinct time intervals. The MRS method provides direct insights into the primary factors driving changes in resistivity, thus synergizing the results of both methods. Although the SP method has a relatively limited dataset, it nonetheless captures the trend of groundwater infiltration during the mining process.

Ground surveys uncovered numerous ground fractures, indicating that subsidence of the overlying bedrock caused by mining activities has impacted the surface. TL-ERT revealed an increase in resistivity in the shallow subsurface layers and a decrease in the deeper layers. This implies that as mining progresses, fractures gradually develop and connect to the shallow aquifer layers, creating pathways for water infiltration and facilitating the continuous downward flow of groundwater. Combining these findings with the results of the MRS method, the observed increase in water content in the deeper subsurface layers aligns with the corresponding decrease in resistivity at those depths. We propose that the extensively developed water-conducting fracture zone extends to depths of approximately 60–82 m, with the groundwater infiltration zone situated at depths of approximately 34–45 m and elevations at approximately 1300 m. The study area is located in a western inland arid–semi-arid region. Although the numerical changes in water content (resulting in resistivity changes of 5–10 Ω·m) may appear relatively modest, the persistent loss of groundwater has irreversible ecological implications for the local environment.

The integration of the ERT, MRS, and SP techniques offers a comprehensive insight into the variations in subsurface water content in the research area. This approach proves to be efficient and economical, providing a geophysical foundation for local ecological management, restoration efforts, and water resource conservation in coal mining activities. Furthermore, it serves as a valuable reference for similar case studies and research initiatives.

## Data Availability

Data available on request from the corresponding author.
